# Tenecteplase for Ischemic Stroke due to Large Vessel Occlusion at 4.5 to 24 Hours: A Meta‐Analysis of Randomized Controlled Trials

**DOI:** 10.1002/brb3.71037

**Published:** 2025-11-21

**Authors:** Shuangzhe Wu, Liyuan Wang, Zhixin Cao, Manjun Hao, Na Wu, Kejia Shen, Yizhe Tang, Yige Zhang, Yujie Ma, Zhixin Li, Yunyun Xiong

**Affiliations:** ^1^ Department of Neurology, Beijing Tiantan Hospital Capital Medical University Beijing China; ^2^ China National Clinical Research Center for Neurological Diseases Beijing China; ^3^ Chinese Institute for Brain Research Beijing China

**Keywords:** 4.5 to 24 h, ischemic stroke, large vessel occlusion, tenecteplase

## Abstract

**Background:**

Whether to use tenecteplase for acute ischemic stroke patients in the extended window has so far been little studied.

**Methods:**

In this meta‐analysis, we included all the randomized controlled trials comparing tenecteplase with control at 4.5 to 24 h after the last known well of acute ischemic stroke with large vessel occlusion. A systematic search was performed in PubMed, Web of Science, Embase, and Cochrane Central Register of Controlled Trials databases, up to 12th June, 2024. Our primary outcome was an excellent functional outcome with a modified Rankin scale score (mRS) of 0–1 at 90 days. Our safety outcomes included mRS 5–6 at 90 days, mortality within 90 days, symptomatic intracranial hemorrhage and parenchymal hematoma Type 2.

**Results:**

Ultimately, three studies involving 1198 patients were included in the pooled analysis, where tenecteplase exhibited a higher rate of mRS 0–1 at 90 days (OR, 1.36; 95%CI, 1.07–1.75; *p* = 0.01) and recanalization (OR, 3.30; 95%CI, 1.59–6.84; *p* = 0.001). For other outcomes, no significant differences were found between groups.

**Conclusions:**

This meta‐analysis of randomized controlled trials manifested that tenecteplase within a 4.5‐ to 24‐h time window of large vessel occlusion stroke was superior to control for an excellent functional outcome (mRS 0–1) without safety concerns.

## Graphical abstract

**Question**: Is it safe and effective to use tenecteplase for acute ischemic stroke patients beyond 4.5 h?

**Finding**: This meta‐analysis of randomized controlled trials manifested that tenecteplase administered within 4.5 to 24 h for large vessel occlusion stroke was superior to control for an excellent functional outcome without safety concerns.

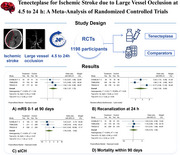

**Abbreviations**
AIS: acute ischemic stroke
AOL: arterial occlusive lesion
CHABLIS‐T: Chinese Acute Tissue‐Based Imaging Selection for Lysis in Stroke ‐Tenecteplase
CI: confidence interval
DT: delay time
EVT: endovascular thrombectomy
GDT: GRADEpro guideline development tool
GRADE: Grading of Recommendations, Assessment, Development, and Evaluation
IVT: intravenous thrombolysis
LVO: large vessel occlusion
mRS: modified Rankin scale
mTICI: modified thrombolysis in cerebral infarction
NIHSS: National Institutes of Health Stroke Scale
OR: odds ratio
PH2: parenchymal hematoma type 2
PRISMA: preferred reported items for systematic reviews and meta‐analyses
ReML: restricted maximum likelihood
SE: standard errors
sICH: symptomatic intracranial hemorrhage
TIMELESS: Thrombolysis in Imaging Eligible, Late Window Patients to Assess the Efficacy and Safety of Tenecteplase
TRACE‐III: Tenecteplase Reperfusion Therapy in Acute Ischemic Cerebrovascular Events‐III

## 1 Introduction

As the leading cause of global disability and death, acute ischemic stroke (AIS) has seen a substantial increase in its burden from 1990 to 2021, especially in low‐ and middle‐income countries (Feigin et al. 2024; Tu et al. 2023). In accordance with prevailing guidelines, intravenous thrombolysis (IVT) with alteplase is the standard medical treatment for AIS patients who arrive within 4.5 h after stroke onset (Powers et al. 2019). Tenecteplase is a genetically engineered variant of alteplase, which has proven pharmacological advantages over alteplase enabling its bolus administration instead of infusion, and is also recommended in the 2019 American Heart Association/American Stroke Association Guidelines as a potential substitute for alteplase in specific AIS patients (Powers et al. 2019). In succession, ample evidence, including multicenter, Phase III, large randomized controlled trials, has proven that intravenous tenecteplase at a dosage of 0.25 mg/kg is non‐inferior to alteplase when administered within 4.5 h (Menon et al. 2022; Muir et al. 2023; Wang et al. 2023). Furthermore, the latest meta‐analysis (Alamowitch et al. 2023) has shown that tenecteplase is superior to alteplase in the subgroup of patients with stroke due to large vessel occlusion (LVO).

For LVO patients, thrombectomy is indispensable after the administration of IVT to attain further reperfusion in a 4.5‐h window. In the late window beyond 4.5 h, with scarce evidence upholding IVT, the efficacy of IVT is deemed to be further weakened, and thrombectomy alone becomes the first‐line therapy for LVO patients. This diminishment in IVT efficacy results primarily from its inability to fully resolve large thrombi in the large vessel and the heightened risk of symptomatic intracranial hemorrhage (sICH) when it's administered beyond 4.5 h (Emberson et al. 2014). Considering both the pharmacological and clinical superiority of tenecteplase over alteplase in LVO patients, it's hypothesized that IVT with tenecteplase might overcome such a hindrance and benefit LVO patients in the late window, which is specifically important to low or middle‐income countries where thrombectomy might not be accessible. Recently published three large randomized controlled trials, the Chinese Acute Tissue‐Based Imaging Selection for Lysis in Stroke—Tenecteplase II (CHABLIS‐T II) study (Cheng et al. 2025), the Thrombolysis in Imaging Eligible, Late Window Patients to Assess the Efficacy and Safety of Tenecteplase (TIMELESS) study (Albers et al. 2024), and the Tenecteplase Reperfusion Therapy in Acute Ischemic Cerebrovascular Events‐III (TRACE‐III) study (Xiong et al. 2024), compared 0.25 mg/kg tenecteplase with control, for patients with LVO of the internal carotid artery, or middle cerebral artery M1 or M2 segments, presenting 4.5 to 24 h, and who had salvageable brain tissue confirmed by perfusion imaging, yet they demonstrated inconsistent results. Hence, a systematic understanding of this realm is urgently required.

We performed this meta‐analysis to establish a comprehensive profile regarding the efficacy and safety of tenecteplase initiated 4.5 to 24 h for LVO patients with salvageable brain tissue.

## 2 Methods

Our study was performed under the guidelines of the Preferred Reported Items for Systematic Reviews and Meta‐Analyses (PRISMA) statement (Page et al. 2021) and was registered with the International Prospective Register of Systematic Reviews (PROSPERO ID CRD42024551479). The corresponding author is responsible for the integrity and accuracy of the data presented in this manuscript. The data that support the findings of this study are available from the corresponding author upon reasonable request.

### 2.1 Search Strategies

We searched PubMed, Web of Science, Embase, and Cochrane Central Register of Controlled Trials databases with a comprehensive search strategy (Supporting Information) to identify eligible studies, without language restrictions up to June 12, 2024. We also searched all the references of the included studies for further identification, and added the data of the TRACE‐III study after its final online publication on June 15, 2024.

### 2.2 Eligibility Criteria

Randomized controlled trials were included if they enrolled patients aged 18 years or older who were diagnosed with AIS due to LVO and who received tenecteplase or comparators 4.5 to 24 h after symptom onset or they were last known well. Studies were excluded if they involved patients with minor stroke or transient ischemic attack, multiple arterial occlusions, or large infarct cores.

### 2.3 Study Screening and Data Extraction

Two reviewers (S.W. and L.W.) independently screened the titles and abstracts of all the studies retrieved from the four databases after eliminating duplicate records. Subsequently, the full texts of potentially eligible studies were reviewed, and studies were ultimately included if they precisely met our predetermined eligibility criteria. During the whole screening process, any discrepancy was judged by a third member (Y.X.) and resolved by further discussion.

The same two reviewers independently abstracted data from included trials using a prespecified data extraction form containing the following information: (1) basic information of the trials including the publication year, study setting, study type, sample size, and follow‐up duration; (2) characteristics of participants including the eligibility criteria (both clinical and imaging criteria), the mean or median age according to different studies, the proportion of female, and median National Institutes of Health Stroke Scale (NIHSS) scores; (3) features of the study procedure including the proportion of patients undergoing endovascular thrombectomy (EVT), the proportion of witnessed stroke, the median time from last known well to the administration of tenecteplase, and details of study interventions and comparators; (4) data for risk of bias assessment; (5) results of selected outcomes in included studies. Eventually, agreements were reached by group discussion on all elements.

### 2.4 Risk of Bias Assessment

Two review authors (S.W. and L.W.) independently assessed risk of bias for included studies using the Version 2 Cochrane risk‐of‐bias assessment tool (RoB 2) (Sterne et al. 2019). Implementing prearranged algorithms embedded in the RoB 2, we rated bias in 5 domains that are currently deemed to affect the quality of randomized controlled trials, as 3 levels defined as low risk of bias, some concerns, or higher risk of bias. Judgment by a third member (Y.X.) was applied if there was any inconsistency.

### 2.5 Study Outcomes

For this meta‐analysis, the prespecified primary outcome was the proportion of patients with an excellent functional outcome (defined as a modified Rankin scale [mRS] score of 0–1) at 90 days. Secondary outcomes included a good functional outcome (defined as an mRS score of 0–2) at 90 days, the ordinal distribution of mRS scores at 90 days, recanalization at 24 h, and reperfusion at 24 h. Safety outcomes were mRS 5–6 at 90 days, mortality within 90 days, sICH, and parenchymal hematoma Type 2 (PH2).

### 2.6 Statistical Analysis

This meta‐analysis is aligned with the Cochrane Handbook for Systematic Reviews of Interventions (Higgins et al. 2023). We entered data of predetermined outcomes into the Stata/MP 17.0 software for data synthesis and presentation. We calculated odds ratio (OR) estimates and 95% confidence interval (CI) after pooling event and total counts of dichotomous outcomes, using the Mantel–Haenzel method in the fixed‐effects model, and the DerSimonian and Laird inverse variance method in the random‐effects model. We meta‐analyzed log ORs and their standard errors (SEs) for ordinal outcomes, using the generic inverse‐variance method, and eventually reported the pooled OR and its 95% CI. The Cochran *Q* test and *I* ^2^ statistics were used to evaluate statistical heterogeneity. A fixed‐effects model would be applied if *I* ^2^ was over 50% and a random‐effects model would be applied if *I* ^2^ was no more than 50%.

Exploratory univariate meta‐regression analyses using the restricted maximum likelihood (ReML) method to investigate the association between different study characteristics, including age, study settings, sample size, rates of thrombectomy, imaging criteria, and outcome definitions. The treatment effects estimated by log ORs of outcomes with *I* ^2^ over 50% were done to help explain the substantial heterogeneity. In sensitivity analyses to further validate the study robustness, we conducted a repeat meta‐analysis, including only Phase 3 trials and synthesized data from patients who did not undergo thrombectomy on mRS distribution. Furthermore, we performed subgroup analyses for the primary outcome (mRS 0–1) based on occlusion site (ICA vs. others), time window (4.5–9 h vs. 9–24 h), and EVT implementation (yes vs. no).

### 2.7 Quality Assessment

Two reviewers (S.W. and L.W.) independently judged the certainty of evidence employing the Grading of Recommendations, Assessment, Development, and Evaluation (GRADE) method for an excellent functional outcome, a good functional outcome, recanalization, reperfusion, and all the safety outcomes, while using the online GRADEpro guideline development tool (GDT) to prepare the summary of findings table, which described the magnitudes of treatment effects, the amount of available studies, as well as the certainty in the body of evidence. Any divergence was addressed by a third member (Y.X.).

## 3 Results

### 3.1 Characteristics of Included Studies

**FIGURE 1:**
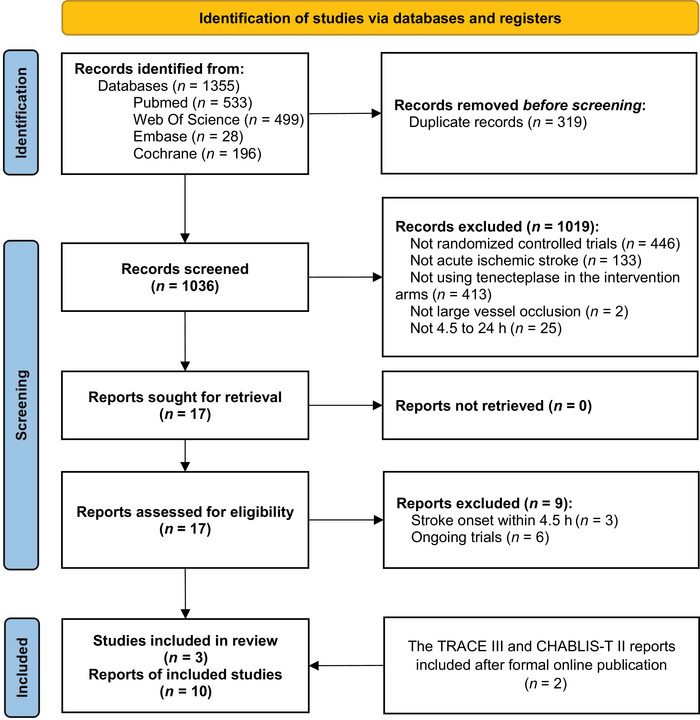
Flow diagram.

After thorough searching, 1355 records were identified, and 17 records were included in the first step of screening. In the second step, after retrieving and screening the full texts, we excluded three reports from two studies that focused on stroke within 4.5 h,(Bivard et al. 2017; Roaldsen et al. 2023) and six reports from four ongoing trials (Nct 2020, 2021, 2024; Pandit et al. 2024). Ultimately, three randomized controlled trials were included: the CHABLIS‐T II study (Cheng et al. 2025), the TIMELESS study (Albers et al. 2024), and the TRACE‐III study (Xiong et al. 2024). Details on the results of search and the exclusion profile are shown in Figure 1.

**FIGURE 2:**
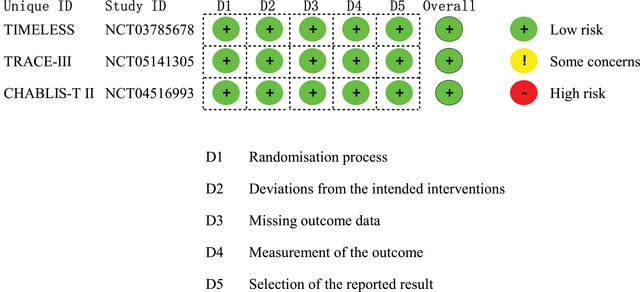
Risk of bias assessment.

A total of 1198 patients were included in the pooled analysis, with 603 assigned to intravenous tenecteplase and 595 to control. Among these three multicenter randomized controlled trials, the TIMELESS study was double‐blind, while the others were conducted with an open‐label design and blinded‐endpoint assessment. Although the three studies shared similar eligibility criteria, the TRACE‐III study excluded patients intending to proceed to EVT, and the CHABLIS‐T II study differed in the target mismatch profile on CT perfusion or MR perfusion, among other slight differences. Moreover, the TIMELESS study included the oldest patients with the highest median baseline NIHSS scores of the three studies. All studies used tenecteplase 0.25 mg/kg, compared with placebo in the TIMELESS study and best/standard medical treatment in the others. Other essential information for each study is displayed in Table S1. All studies were judged to have low risk of bias (Figure 2).

### 3.2 The Primary Outcome

**FIGURE 3:**
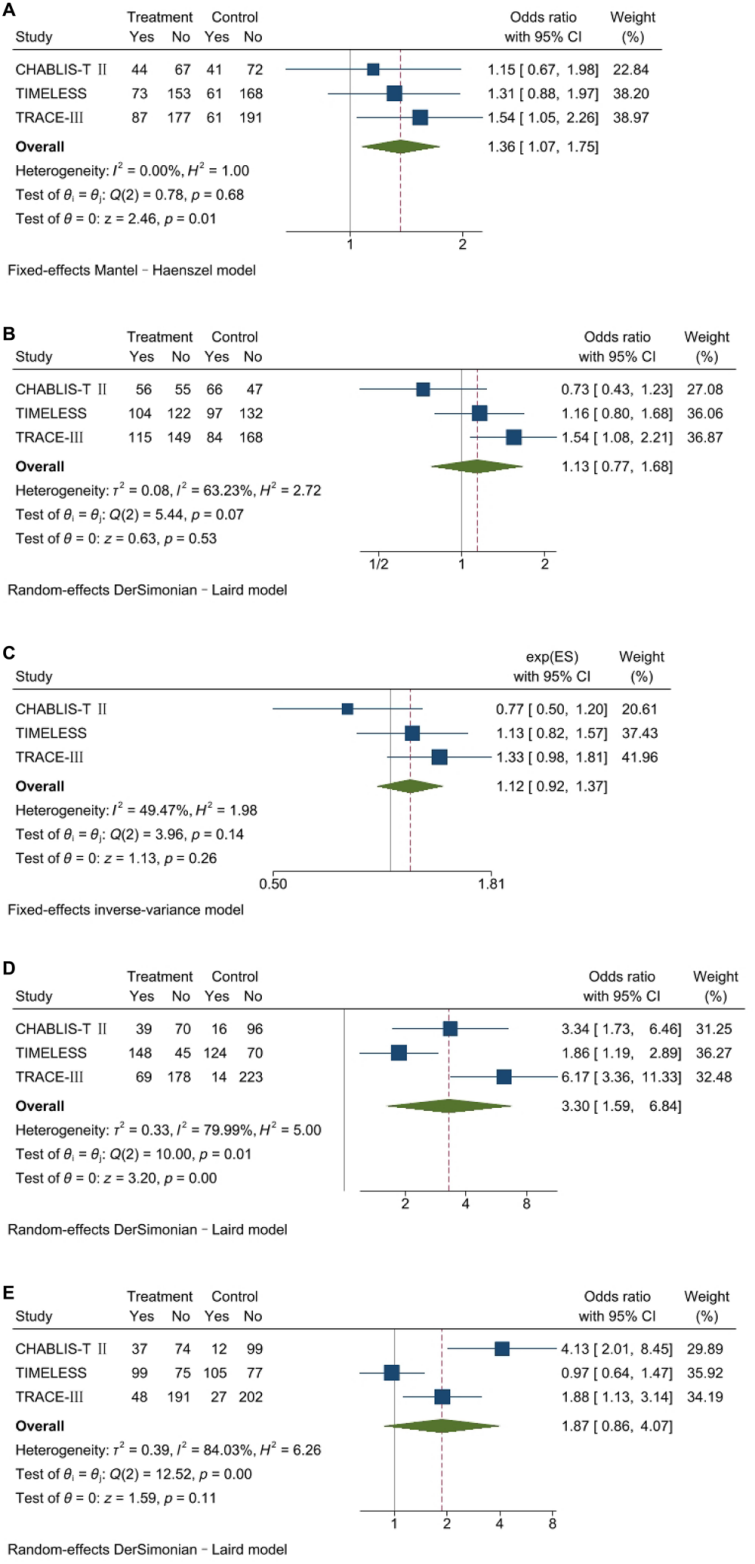
Meta‐analyses for efficacy outcomes. Forest plots showing the relation of tenecteplase versus control with (A) an excellent functional outcome defined as an mRS score of 0–1 at 90 days, (B) a good functional outcome defined as an mRS score of 0–2 at 90 days, (C) the ordinal distribution of mRS scores at 90 days, (D) recanalization at 24 h, and (E) reperfusion at 24 h. The sizes of the data markers correspond to the weight of each study in the meta‐analysis. The diamond represents the pooled overall estimate. CI, confidence interval; mRS, modified Rankin scale.

The pooled analysis for the excellent functional outcome comprised a total of 1195 patients, where 33.9% in the tenecteplase group and 27.4% in the control group reached mRS 0–1 at 90 days, and showed a statistically significant difference in favor of tenecteplase (OR, 1.36; 95%CI, 1.07–1.75; *p* = 0.01; *I* ^2 ^= 0%; Figure 3).

### 3.3 Secondary Outcomes

We predefined four secondary efficacy outcomes: an mRS score of 0–2 at 90 days, the mRS distribution at 90 days, recanalization, and reperfusion within different time frames among studies, which involved 1195, 1195, 1092, and 1046 patients in the synthesis of results, respectively. Patients in the tenecteplase group had significantly higher recanalization rates than those in the control group, whereas no significant differences were observed in other outcomes. For recanalization and reperfusion evaluation, the three studies varied in outcome definitions. The TIMELESS study and TRACE‐III study both evaluated complete recanalization at 24 h using arterial occlusive lesion (AOL) scores of three. However, the CHABLIS‐T II study assessed recanalization on 4‐ to 6‐h CTA for patients not receiving EVT or on initial angiogram before EVT for patients receiving EVT, using Thrombolysis in Myocardial Infarction scale scores ≥ 2. The same two trials collected data on reperfusion at 24 h, defined as > 90% reduction in the penumbra volume on CTP (*T*_max_ > 6 s). Yet, the CHABLIS‐T II study combined major reperfusion with an absence of sICH at 24–48 h as a composite outcome, where major reperfusion was defined as ≥ 50% reperfusion in the ischemic territory on 4‐ to 6‐h CTP without EVT or on initial catheter angiography (modified thrombolysis in cerebral infarction [mTICI] scores 2b‐3) prior to EVT. Definitions of the study outcomes and the corresponding results are detailed in Table S2 and Figure 3, respectively.

### 3.4 Safety Outcomes

**FIGURE 4:**
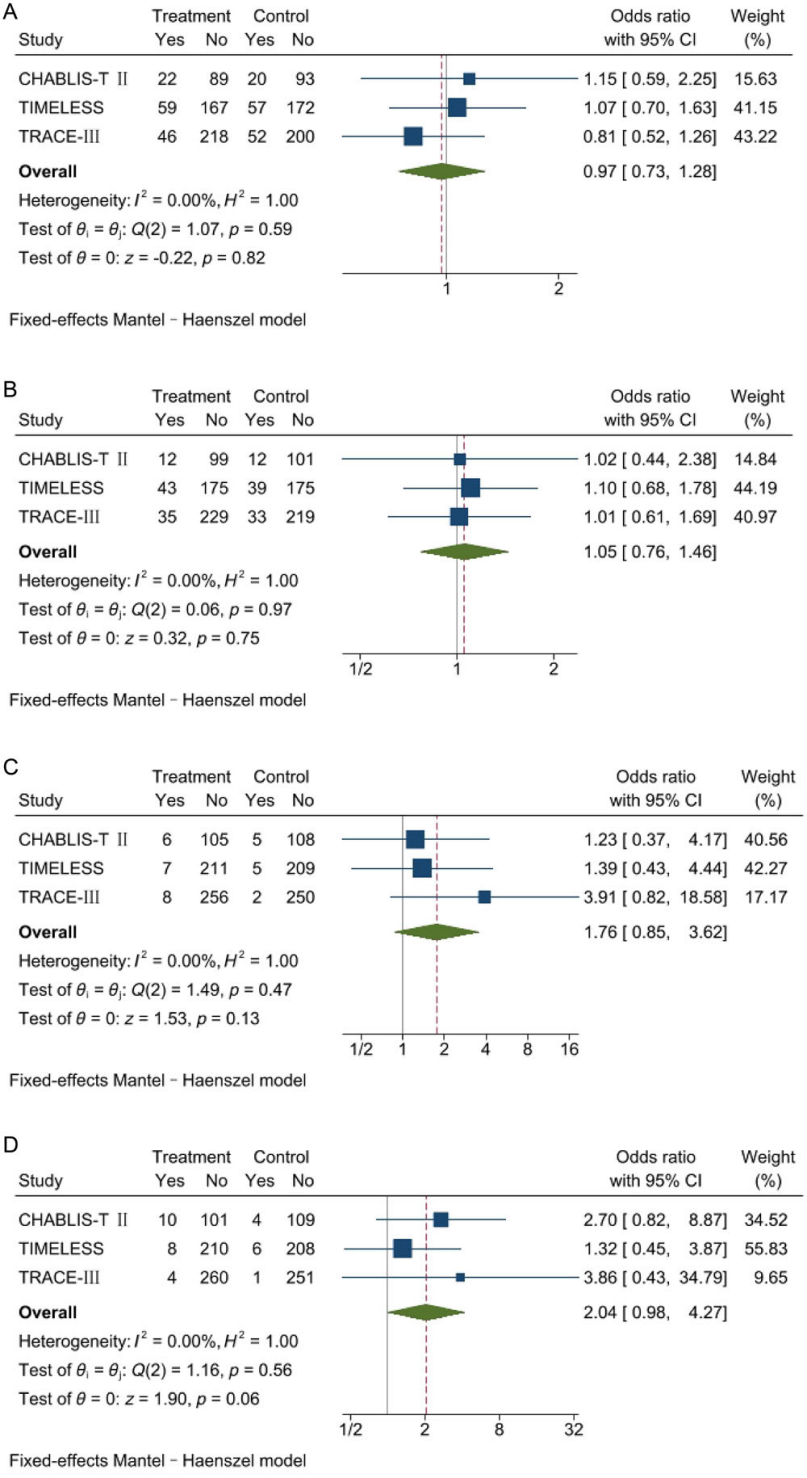
Meta‐analyses for safety outcomes. Forest plots showing the relation of tenecteplase versus control with (A) mRS 5–6 at 90 days, (B) mortality within 90 days, (C) sICH, and (D) PH2. The sizes of the data markers correspond to the weight of each study in the meta‐analysis. The diamond represents the pooled overall estimate. CI, confidence interval; mRS, modified Rankin scale; PH2, parenchymal hematoma type 2, sICH, symptomatic intracranial hemorrhage.

There were no significant differences with respect to safety outcomes between tenecteplase and control. Among 1195 patients, the proportion of patients with mRS 5–6 was 21.1% in the tenecteplase group and 21.7% in the control group (OR, 0.97; 95%CI, 0.73–1.28; *p* = 0.82; *I* ^2 ^= 0%; Figure 4). Composed of 1172 patients, mortality within 90 days was 15.2% and 14.5% (OR, 1.05; 95%CI, 0.76–1.46; *p* = 0.75; *I* ^2 ^= 0%; Figure 4). sICH was assessed within 36 h in the TIMELESS study and TRACE‐III study, or at 24–48 h in the CHABLIS‐T II study. In total, 1172 patients were included, and 3.5% versus 2.1% had sICH respectively in the two groups (OR, 1.76; 95%CI, 0.85–3.62; *p* = 0.13; *I* ^2 ^= 0%; Figure 4). For PH2, assessment time varied from 24–48 h in the CHABLIS‐T II study, 36 h in the TRACE‐III study, to 72 h in the TIMELESS study. Three studies reported data for 1172 patients, among whom 3.7% versus 1.9% experienced PH2 in the tenecteplase and control groups, respectively (OR, 2.04; 95%CI, 0.98–4.27; *p* = 0.06; I^2 ^= 0%; Figure 4).

### 3.5 Meta‐Regression, Sensitivity, and Subgroup Analysis

In the meta‐regression process, significant associations were recognized between the proportion of mRS 0–2 and the study sample size, between recanalization rates and sample size, between recanalization rates and outcome definitions, as well as between reperfusion rates and patient age (Table S3). In the sensitivity analysis, there was a significant difference between groups in mRS 0–2 after removing the Phase 2b study CHABLIS‐T II, with parallel results in other outcomes (Figures S1and S2). Additionally, no difference was uncovered in the mRS distribution after excluding patients who underwent thrombectomy (Figure S3). In subgroup analyses of the primary outcome stratified by occlusion site, time window, and thrombectomy implementation, tenecteplase was associated with a significantly higher rate of mRS 0 to 1 compared with control in the subgroup without EVT (OR, 1.56; 95% CI, 1.10–2.21; *p* = 0.01; *I* ^2^ = 0%; interaction *p* = 0.04; Figure S6), whereas no significant differences were observed in the other subgroups (Figures S4–S6).

### 3.6 GRADE of Evidence and Summary of Findings

For the quality of evidence according to the GRADE criteria in our results, high certainty was appraised for the primary outcome of mRS 0–1, with moderate certainty for all the safety outcomes and low or very low certainty for other outcomes assessed. The summary of findings is presented in Tables S4 andS5.

## 4 Discussion

In view of the lack of evidence on the administration of tenecteplase in the late window, our study compared tenecteplase with standard medical treatment or placebo within 4.5 to 24 h after symptom onset or last known well of AIS due to LVO. In this meta‐analysis, we found that tenecteplase was superior to control regarding an excellent functional outcome with an mRS score of 0–1 and the rate of recanalization. There was no significant difference with regard to safety outcomes.

Preferable results were demonstrated for tenecteplase when compared with alteplase in the extended window. Our results showed similar efficacy with a previous meta‐analysis of individual patient data from the EXTEND (Ma et al. 2019), ECASS‐4 (Ringleb et al. 2019), and EPITHET (Davis et al. 2008) trials, which compared alteplase with placebo among AIS patients presenting at 4.5 to 9 h after symptom onset (Bruce C. V. Campbell et al. 2019). In its subgroup analysis, higher odds were demonstrated for patients with LVO, also comparable with what this analysis discovered for LVO patients presenting at 4.5 to 24 h after last known well. However, the aforementioned analysis only included patients receiving alteplase 4.5 to 9 h, a time frame much shorter than the upper limit of 24 h in our analysis, with a relatively small sample size of 304 in total and 219 in the LVO subgroup, compared to 1198 in our study. Notably, alteplase was found to have a statistically higher risk of sICH than placebo, in contrast to our results that suggested no safety concerns for tenecteplase.

Moreover, we identified a potential tendency for tenecteplase to perform better in lower scores of mRS, as there was no significant difference concerning mRS scores of 0–2 or 5–6. Improvement in the excellent outcome might be counteracted by a potential increase in poor outcomes, leading to similar mRS distribution between groups. In this analysis, the heightened incidence of recanalization did not necessarily translate to a higher incidence of reperfusion.

The TRACE‐III study (Xiong et al. 2024) excluded patients who were scheduled for thrombectomy and unearthed significant treatment effects regarding functional outcomes and no difference in safety outcomes. It strongly substantiated the efficacy of tenecteplase which has been previously concealed by thrombectomy. TIMELESS (Albers et al. 2024) included 77.3% of LVO patients who received thrombectomy and manifested neutral results in both functional and safety outcomes. As TIMELESS mostly took place at EVT‐capable centers, where thrombectomy was initiated promptly after tenecteplase with a median of 15 min, it did not support the interim use of tenecteplase for patients who arrived directly at thrombectomy centers (also known as direct‐to‐mothership pattern). In CHABLIS‐T II (Cheng et al. 2025), 54.5% of LVO patients received thrombectomy, and similar results with TIMELESS were shown. These studies scrutinized particular subsets of the general AIS population to enhance comprehension of tenecteplase administration in diverse settings. Nevertheless, a vast number of patients who initially arrived at primary stroke centers and were subsequently transferred to EVT‐capable centers (also known as drip‐and‐ship pattern) remain unexamined in these trials. Our results appeared to be an intermediate between these trials, with 40.5% of participants having received thrombectomy. Compared with 1.7% in the TRACE‐III study, our analysis demonstrated a rate closer to the real‐world data in countries utilizing stroke bypass protocols with more drip‐and‐ship patients (Asif et al. 2023). By appending more cases with arteries totally or partially reopened by tenecteplase to the TIMELESS study, our analysis potentially simulated an interhospital transfer setting that allowed more time for tenecteplase to function. European Stroke Organization guidelines indicated a knowledge gap that there are no randomized data for patients presenting to a non‐thrombectomy center beyond 4.5 h who are set to undergo thrombectomy and are also candidates for thrombolysis, and failed to reach group consensus on whether to give thrombolysis before thrombectomy (Berge et al. 2021). Our pooled analysis could help fill this knowledge lacuna and provide evidence for the use of tenecteplase in drip‐and‐ship patients. More randomized controlled trials specifically set in the late‐window transfer with administration of tenecteplase are warranted.

Whether to administer thrombolysis before thrombectomy has been much debated. Our meta‐analysis might also facilitate the understanding of bridging therapy in the extended window. Though the IRIS study (Majoie et al. 2023) comparing alteplase plus EVT and EVT alone within 4.5 h, and TIMELESS (Albers et al. 2024) comparing tenecteplase plus thrombectomy versus thrombectomy alone 4.5 to 24 h failed to attest any superiority in functional outcomes, the EXEND‐IA TNK study(B. C. V. Campbell et al. 2018) that compared tenecteplase versus alteplase before thrombectomy within 4.5 h showed a significant difference in mRS distribution. TIMELESS and EXTEND‐IA TNK both used tenecteplase in the intervention group, yet there were several key variabilities in the study design that the latter study used tenecteplase much earlier, didn't have any delay in thrombectomy between groups, and gave tenecteplase more time (median, 43 min; IQR, 25–57) to act before thrombectomy. Considering that tenecteplase remains effective beyond 4.5 h as verified by our results, and that the delay in TIMELESS is relatively acceptable, we inferred that in the extended window it's better to use tenecteplase alone or to fill the reperfusion treatment vacancy during transfer so that tenecteplase could have more time to work, either to provide complete reperfusion, or at least some microvascular reperfusion, that might help restrain the unpredictable infarct growth during patient transfer. Previous studies discerned that tenecteplase showed benefits within at least 2 h and 20 min prior to thrombectomy (Kaesmacher et al. 2024), which allowed an adequate time delay for transfer.

Limitations in this analysis are as follows. First, clinical heterogeneity might arise from different study designs and different baseline characteristics. For instance, TRACE‐III (Xiong et al. 2024) excluded thrombectomy, TIMELESS (Albers et al. 2024) included older patients, CHABLIS‐T II (Cheng et al. 2025) applied distinct perfusion mismatch criteria while also including anterior cerebral artery occlusions. However, we only included randomized controlled trials that have already assessed group balance, thereby reducing the influence of these potential confounders. It should be noted that all three studies shared key inclusion criteria including adult patients, NIHSS ≥ 5, 4.5 to 24 h window, pre‐stroke mRS 0 to 2, and anterior circulation occlusion. Both mismatch criteria used in these trials were proven effective for penumbral selection (Albers et al. 2018; Ma et al. 2019). Second, substantial statistical heterogeneity was observed for the outcomes of recanalization, reperfusion, and mRS 0–2. Our exploratory meta‐regression analysis further indicated that differences in outcome definitions, sample size and mean age across studies were sources of this heterogeneity. Of note, the CHABLIS‐T II study used different definitions for reperfusion, recanalization, sICH, and PH2, causing difficulty in pooling the data, so it's recommended to interpret these outcomes in our analysis with caution. However, we used various methods to address relevant statistical heterogeneity, comprising a random‐effects model to account for variability across studies, and a sensitivity analysis, which showed consistent results for all outcomes except mRS 0–2. Despite considerable heterogeneity, sensitivity analysis further supported the robustness of our results by excluding the CHABLIS‐T II study, which differed most markedly from the other two studies in outcome definitions and sample size. Third, despite having a relatively large sample size of 1198, our analysis only consisted of three trials, impeding the implementation of subgroup analysis, more sensitivity analyses, and powerful meta‐regression to dig into the origin of the above heterogeneity, prove reliability, or suggest avenues for future investigation. Besides, further assessment of publication bias, including funnel plots and Egger's or Begg's test, was unavailable with only three included studies. Several ongoing trials (NCT06221371, NCT05105633, CTRI/2022/03/040718) evaluating tenecteplase for LVO in extended time windows are expected to provide larger, more geographically diverse samples for future meta‐analyses. Extending the time window for tenecteplase treatment holds particular value for low‐ and middle‐income countries due to limited EVT access, where further studies are needed to confirm the applicability of our results. Moreover, real‐world evidence is warranted to reinforce the feasibility and safety of administering tenecteplase within 4.5 to 24 h of stroke onset in routine clinical practice.

## 5 Conclusions

We performed the first meta‐analysis of randomized data to investigate the efficacy and safety of tenecteplase outside of the standard time window and yielded positive results in favor of tenecteplase if patients presented 4.5 to 24 h and had salvageable brain tissue confirmed by perfusion mismatch for AIS due to anterior circulation LVO.

## Author Contributions

Concept and design: Yunyun Xiong and Shuangzhe Wu. Drafting of the manuscript: Shuangzhe Wu, Liyuan Wang, and Yunyun Xiong. Critical revision of the manuscript for important intellectual content: Yunyun Xiong. Administrative, technical, or material support: Zhixin Cao, Manjun Hao, and Na Wu. Supervision: Yunyun Xiong. All authors acquired, analyzed, interpreted the data, reviewed and edited the manuscript, and approved the final version of the manuscript.

## Funding

This work was supported by the Beijing Municipal Science Fund for Distinguished Young Scholars (JQ24058) and the National Natural Science Foundation (grant number 82571467).

## Conflicts of Interest

The authors have nothing to report.

## Supporting information



The comprehensive search strategy
Table S1‐S5
Figure S1‐S6

## Data Availability

The data that support the findings of this study are available from the corresponding author upon reasonable request.
