# Supplementary material for: Tenecteplase for Ischemic Stroke due to Large Vessel Occlusion at 4.5 to 24 Hours: A Meta‐Analysis of Randomized Controlled Trials
Source: Brain Behav. 2025 Nov 21;15(11):e71037. doi: 10.1002/brb3.71037 (PMC12638445; doi:10.1002/brb3.71037)
Supplement: Supplementary file 1 — The comprehensive search strategy Table S1‐S5 Figure S1‐S6 [file BRB3-15-e71037-s001.docx]

**Supplemental Material**

**Tenecteplase for Ischemic Stroke at 4.5 to 24 Hours: A Meta-Analysis of Randomized Controlled Trials**

**Table of Contents**

|  | Page |
| --- | --- |
| The comprehensive search strategy. | 3 |
| Figure S1 A sensitivity analysis including only phase 3 trials on efficacy outcomes. | 9 |
| Figure S2 A sensitivity analysis including only phase 3 trials on safety outcomes. | 11 |
| Figure S3 A sensitivity analysis including only patients without thrombectomy of modified Rankin scale score distribution at 90 days. | 13 |
| Figure S4 A subgroup analysis of a modified Rankin scale score of 0-1 based on occlusion sites. | 14 |
| Figure S5 A subgroup analysis of a modified Rankin scale score of 0-1 based on time windows. | 15 |
| Figure S6 A subgroup analysis of a modified Rankin scale score of 0-1 based on thrombectomy implementation. | 16 |
| Table S1 Characteristics of included studies. | 17 |
| Table S2 Outcome definitions of included studies. | 21 |
| Table S3 Univariate meta-regression analyses. | 24 |
| Table S4 Summary of findings for efficacy outcomes. | 25 |
| Table S5 Summary of findings for safety outcomes. | 27 |

**COMPREHENSIVE SEARCH STRATEGY**

**PUBMED**

#1 “ischemic stroke”[mh] OR ischemic stroke OR AIS OR brain infarction OR embolic stroke OR thrombotic stroke OR cerebral ischemia OR brain ischemia

#2 large vessel OR proximal vessel OR major vessel OR large artery OR proximal artery OR major artery OR LVO OR PVO OR internal carotid artery OR middle cerebral artery OR anterior cerebral artery OR basilar artery OR vertebral artery

#3 “beyond 4.5 hours” OR “more than 4.5 hours” OR “exceeding 4.5 hours” OR “over 4.5 hours” OR “longer than 4.5 hours” OR “greater than 4.5 hours” OR “after 4.5 hours” OR “past 4.5 hours” OR “4.5 hours post” OR “following 4.5 hours” OR “elapsed 4.5 hours” OR “4.5 hours later” OR “post 4.5-hour” OR “4.5-hour threshold” OR “4.5 hours and beyond” OR “4.5 hours onward” OR “4.5 hours or more” OR “subsequent to 4.5 hours” OR “past four and a half hours” OR “beyond the standard window” OR “late time window” OR “extended time window” OR “late window” OR “extended window” OR "24 hours”[tiab] OR “twenty-four hours”[tiab] OR “24-hour”[tiab] OR “twenty-four-hour”[tiab] OR “24h”[tiab] OR “24 h”[tiab] OR “twenty-four h”[tiab]

#4 “thrombolytic therapy”[mh] OR thromboly*[tiab] OR fibrinoly*[tiab] OR “tissue plasminogen activator”[mh] OR tissue plasminogen activator[tiab] OR tenecteplase[tiab] OR metalyse[tiab] OR TNK[tiab] OR TNKase[tiab] OR “recombinant human TNK tissue-type plasminogen activator”[tiab]

#5 “randomized controlled trials as topic”[mh] OR “randomized controlled trial”[pt] OR “random allocation”[mh] OR randomized study OR randomized trial OR controlled clinical trial OR controlled clinical study OR random allocation OR placebo OR RCT

#1 AND #2 AND #3 AND #4 AND #5

**Web Of Science**

#1 TS=(ischemic stroke OR AIS OR brain infarction OR embolic stroke OR thrombotic stroke OR cerebral ischemia OR brain ischemia)

#2 TS=(large vessel OR proximal vessel OR major vessel OR large artery OR proximal artery OR major artery OR LVO OR PVO OR internal carotid artery OR middle cerebral artery OR anterior cerebral artery OR basilar artery OR vertebral artery)

#3 TS=(“beyond 4.5 hours” OR “more than 4.5 hours” OR “exceeding 4.5 hours” OR “over 4.5 hours” OR “longer than 4.5 hours” OR “greater than 4.5 hours” OR “after 4.5 hours” OR “past 4.5 hours” OR “4.5 hours post” OR “following 4.5 hours” OR “elapsed 4.5 hours” OR “4.5 hours later” OR “post 4.5-hour” OR “4.5-hour threshold” OR “4.5 hours and beyond” OR “4.5 hours onward” OR “4.5 hours or more” OR “subsequent to 4.5 hours” OR “past four and a half hours” OR “beyond the standard window” OR “late time window” OR “extended time window” OR “late window” OR “extended window”) OR TS=("24 hours” OR “twenty-four hours” OR “24-hour” OR “twenty-four-hour” OR “24h” OR “24 h” OR “twenty-four h”)

#4 TS=(thromboly* OR fibrinoly* OR “tissue plasminogen activator”[mh] OR tissue plasminogen activator OR tenecteplase OR metalyse OR TNK OR TNKase OR “recombinant human TNK tissue-type plasminogen activator”)

#5 TS=(randomized study OR randomized trial OR controlled clinical trial OR controlled clinical study OR random allocation OR placebo OR RCT)

#1 AND #2 AND #3 AND #4 AND #5

**EMBASE**

#1 ‘ischemic stroke’/exp OR ischemic stroke OR AIS OR brain infarction OR embolic stroke OR thrombotic stroke OR cerebral ischemia OR brain ischemia

#2 large vessel OR proximal vessel OR major vessel OR large artery OR proximal artery OR major artery OR LVO OR PVO OR internal carotid artery OR middle cerebral artery OR anterior cerebral artery OR basilar artery OR vertebral artery

#3 ‘beyond 4.5 hours’ OR ‘more than 4.5 hours’ OR ‘exceeding 4.5 hours’ OR ‘over 4.5 hours’ OR ‘longer than 4.5 hours’ OR ‘greater than 4.5 hours’ OR ‘after 4.5 hours’ OR ‘past 4.5 hours’ OR ‘4.5 hours post’ OR ‘following 4.5 hours’ OR ‘elapsed 4.5 hours’ OR ‘4.5 hours later’ OR ‘4.5 hour threshold’ OR ‘4.5 hours and beyond’ OR ‘4.5 hours onward’ OR ‘4.5 hours or more’ OR ‘subsequent to 4.5 hours’ OR ‘past four and a half hours’ OR ‘beyond standard time window’ OR ‘late time window’ OR ‘extended time window’ OR ‘late window’ OR ‘extended window’ OR (‘24 hours’ OR ‘twenty-four hours’ OR ‘24-hour’ OR ‘twenty-four-hour’ OR ‘24h’ OR ‘24 h’ OR ‘twenty-four h’):ti,ab,kw

#4 ‘fibrinolytic therapy’/exp OR (thromboly* OR fibrinoly* OR ‘tissue plasminogen activator’ OR tissue plasminogen activator OR tenecteplase OR metalyse OR TNK OR TNKase OR ‘recombinant human TNK tissue-type plasminogen activator’):ti,ab,kw

#5 ‘randomized controlled trial’/exp OR randomized study OR randomized trial OR controlled clinical trial OR controlled clinical study OR random allocation OR placebo OR RCT

#1 AND #2 AND #3 AND #4 AND #5

**Cochrane Central Register of Controlled Trials databases**

#1 [mh “ischemic stroke”] OR ischemic stroke OR AIS OR brain infarction OR embolic stroke OR thrombotic stroke OR cerebral ischemia OR brain ischemia

#2 large vessel OR proximal vessel OR major vessel OR large artery OR proximal artery OR major artery OR LVO OR PVO OR internal carotid artery OR middle cerebral artery OR anterior cerebral artery OR basilar artery OR vertebral artery

#3 “beyond 4.5 hours” OR “more than 4.5 hours” OR “exceeding 4.5 hours” OR “over 4.5 hours” OR “longer than 4.5 hours” OR “greater than 4.5 hours” OR “after 4.5 hours” OR “past 4.5 hours” OR “4.5 hours post” OR “following 4.5 hours” OR “elapsed 4.5 hours” OR “4.5 hours later” OR “4.5 hour threshold” OR “4.5 hours and beyond” OR “4.5 hours onward” OR “4.5 hours or more” OR “subsequent to 4.5 hours” OR “past four and a half hours” OR “beyond standard time window” OR “late time window” OR “extended time window” OR “late window” OR “extended window” OR ("24 hours" OR "twenty-four hours" OR "24-hour" OR "twenty-four-hour" OR "24h" OR "24 h" OR "twenty-four h"):ti,ab,kw

#4 [mh “thrombolytic therapy”] OR (thromboly* OR fibrinoly* OR “tissue plasminogen activator” OR tissue plasminogen activator OR tenecteplase OR metalyse OR TNK OR TNKase OR “recombinant human TNK tissue-type plasminogen activator”):ti,ab,kw

#5 [mh “randomized controlled trial”] OR [mh “randomized controlled trials as topic”] OR [mh “random allocation”] OR randomized study OR randomized trial OR controlled clinical trial OR controlled clinical study OR random allocation OR placebo OR RCT

#1 AND #2 AND #3 AND #4 AND #5

**
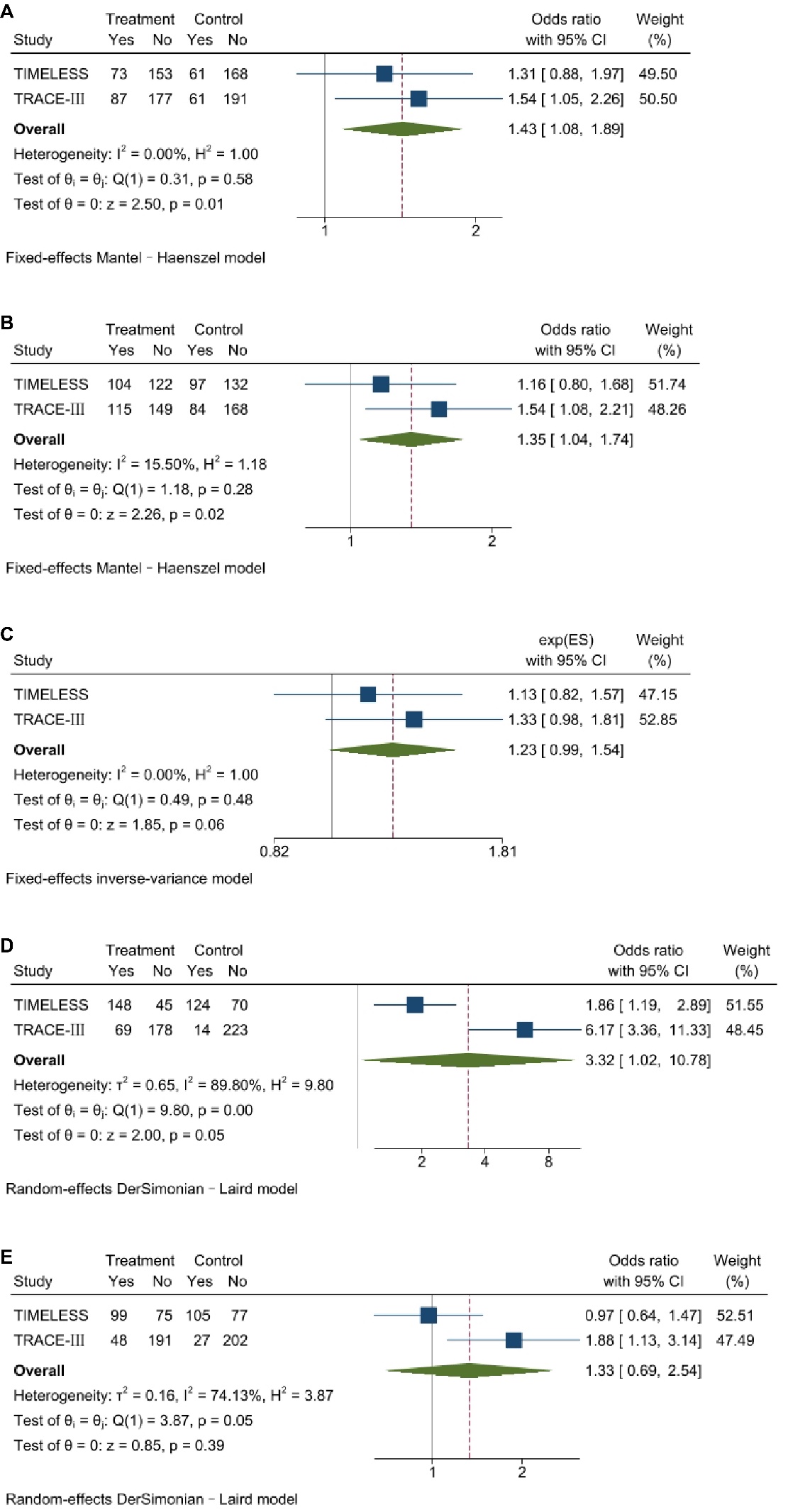
**

**Figure S1** A sensitivity analysis including only phase 3 trials on efficacy outcomes. (A) an excellent functional outcome defined as an mRS score of 0-1 at 90 days, (B) a good functional outcome defined as an mRS score of 0-2 at 90 days, (C) the ordinal distribution of mRS scores at 90 days, (D) recanalization at 24 hours, (E) reperfusion at 24 hours. mRS, modified Rankin scale; CI, confidence interval.


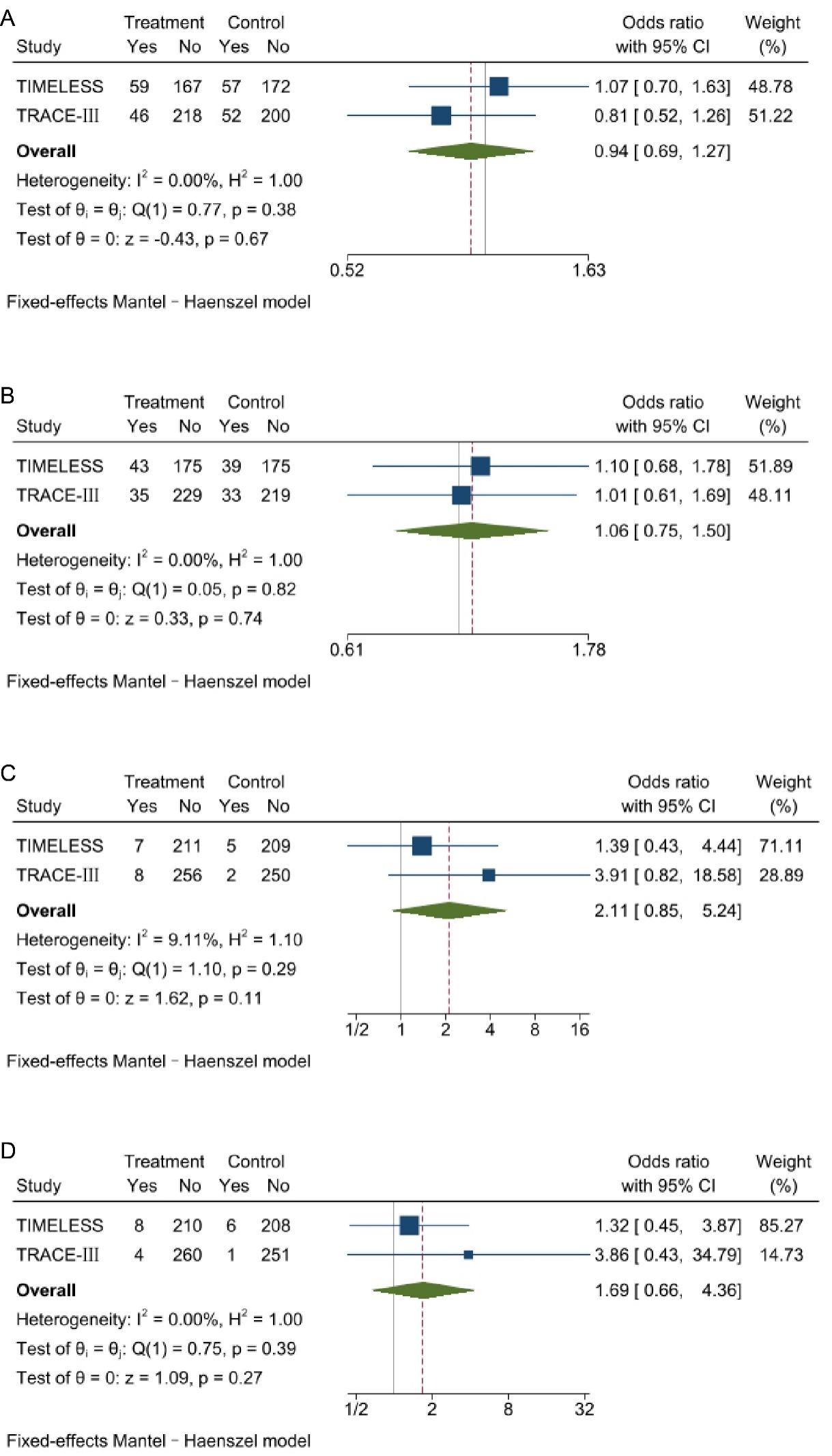


**Figure S2** A sensitivity analysis including only phase 3 trials on safety outcomes. (A) mRS 5-6 at 90 days, (B) mortality within 90 days, (C) sICH, (D) PH2. CI, confidence interval; mRS, modified Rankin scale; sICH, symptomatic intracranial hemorrhage; PH2, parenchymal hematoma type 2.


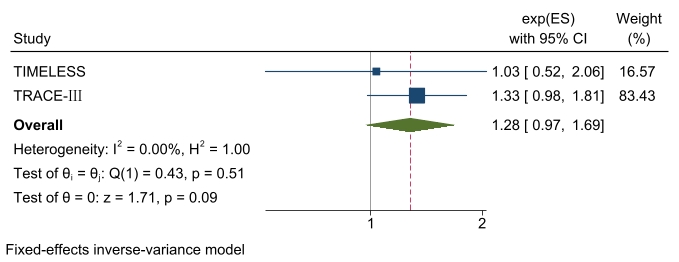


**Figure S3** A sensitivity analysis including only patients without thrombectomy of modified Rankin scale score distribution at 90 days.

**
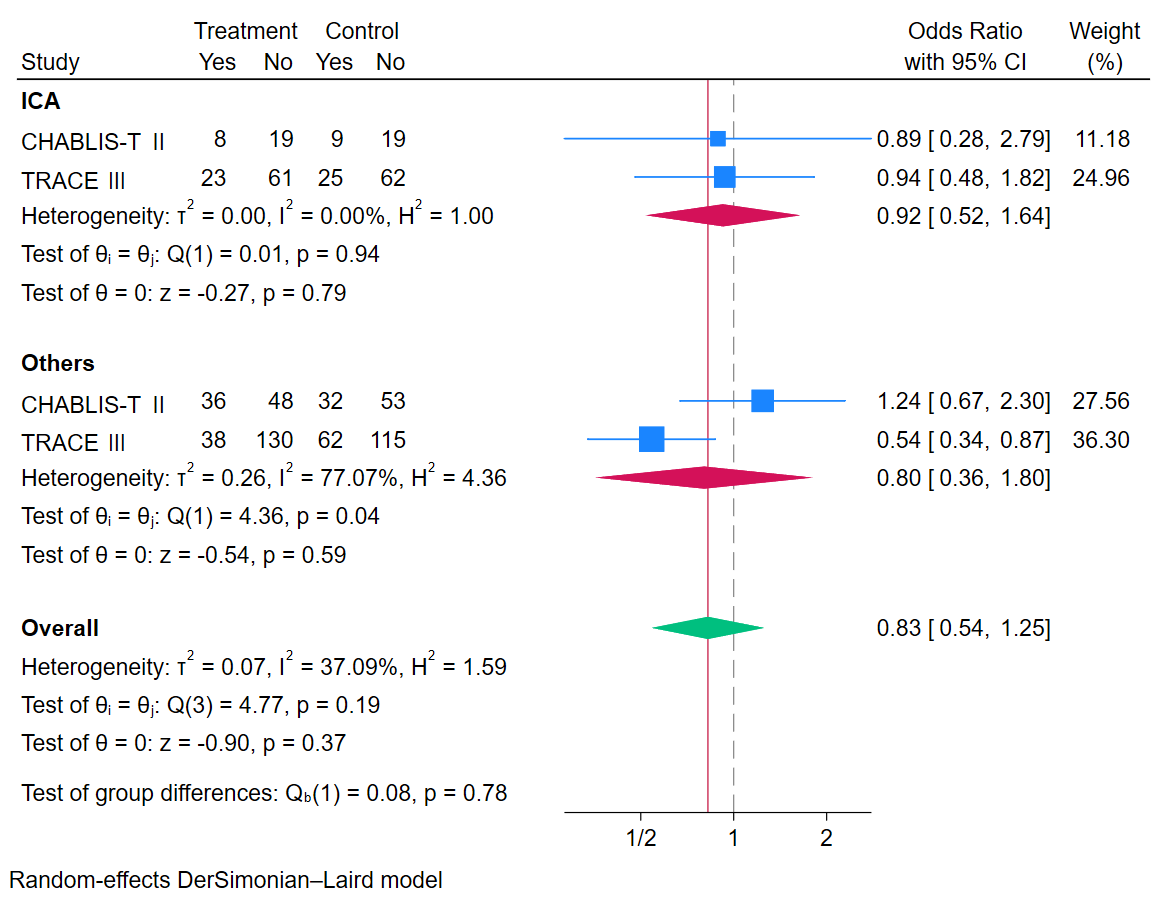
Figure S4** A subgroup analysis of a modified Rankin scale score of 0-1 based on occlusion site.

**
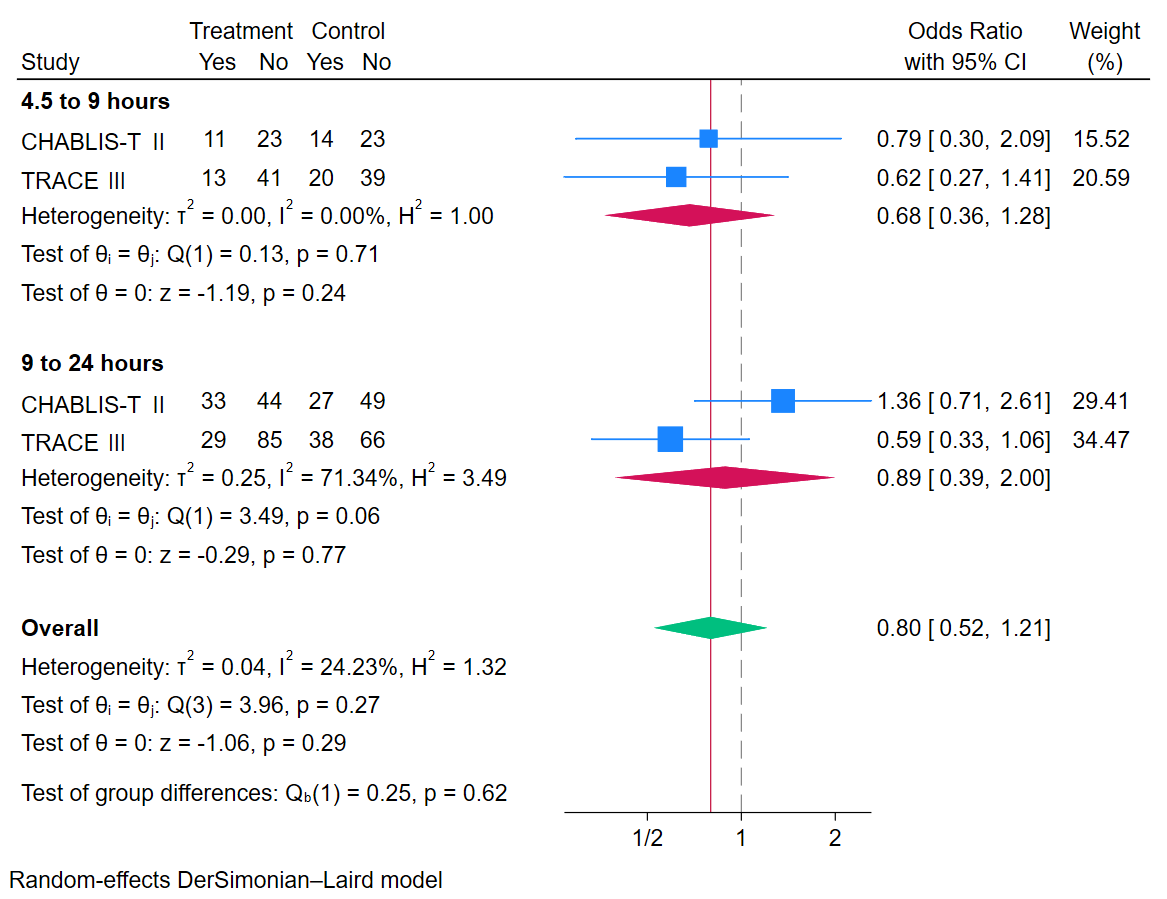
Figure S5** A subgroup analysis of a modified Rankin scale score of 0-1 based on time window.

**
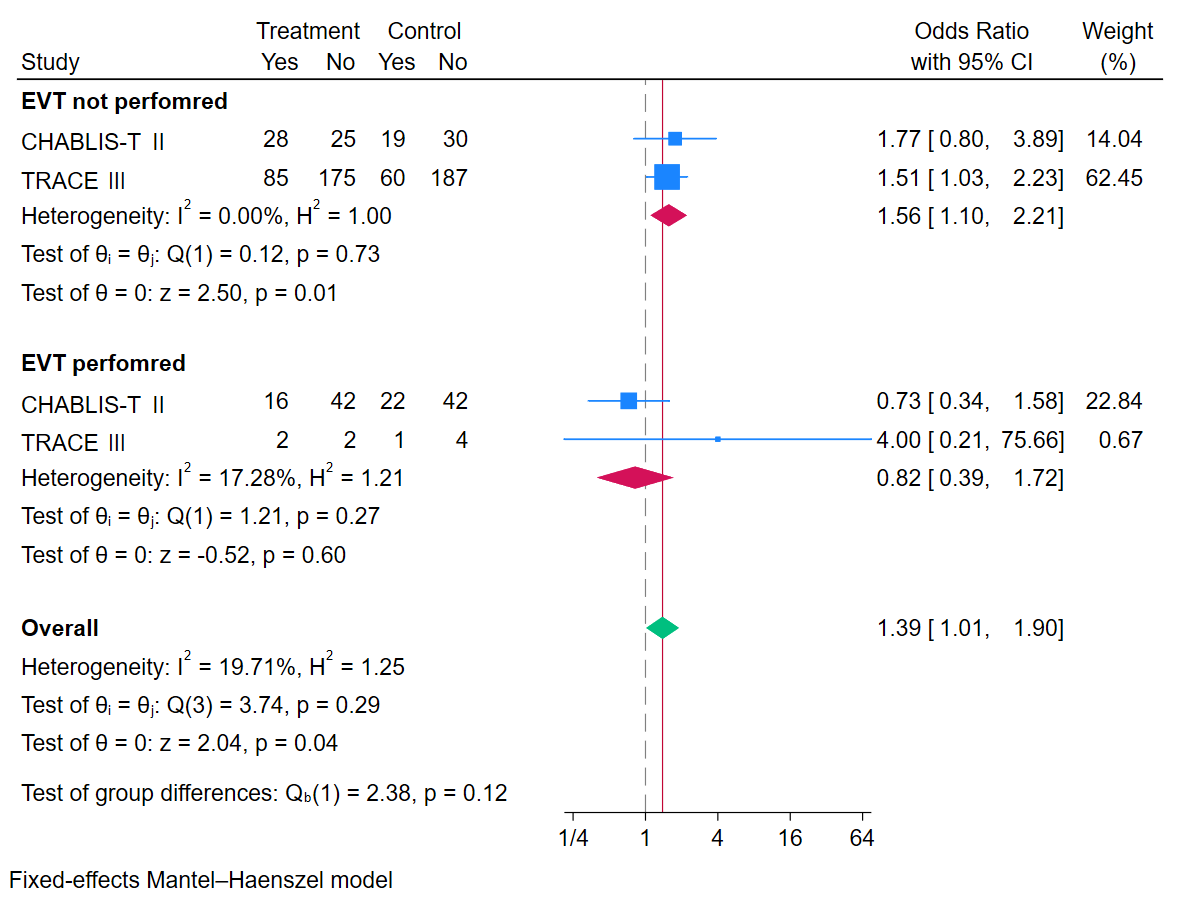
Figure S6** A subgroup analysis of a modified Rankin scale score of 0-1 based on thrombectomy implementation.

**Table S1** Characteristics of included studies.

|  | | | **CHABLIS-T II** | **TIMELESS** | **TRACE-III** |
| --- | --- | --- | --- | --- | --- |
| **Year** | | | 2021.1 to 2023.06 | 2019.3 to 2022.12 | 2022.1 to 2023.11 |
| **Location** | | | 23 centers in China | 112 centers in the USA and Canada | 58 centers in China |
| **Study Type** | | | Multicenter, phase 2b, PROBE, superiority trial | Multicenter, phase 3, double-blind, randomized, placebo-controlled superiority trial | Multicenter, phase 3, PROBE, superiority trial |
| **Sample Size** | *Total* | | 224 | 458 | 516 |
|  | *TNK* | | 111 | 228 | 264 |
|  | *Control* | | 113 | 230 | 252 |
| **Follow-up duration** | | | 90 | 90 | 90 |
| **Study Participants** | *Clinical Criteria* | | 1) age >= 18 years，<= 80  2) Patients presenting with anterior circulation acute ischemic stroke with time from onset to treatment 4.5h to 24h 3) Functionally independent (mRS 0-2) prior to stroke onset 4) Baseline NIHSS >= 6 5) Clinically significant acute neurologic deficit | 1) Age >= 18 years 2) AIS symptom onset (last known well) within 4.5 to 24 hours 3) Signs and symptoms consistent with the diagnosis of an acute anterior circulation ischemic stroke involving occlusion of the ICA, M1, or M2 vessels 4) Functionally independent (mRS 0-2) prior to stroke onset 5) Baseline NIHSS >=5 and that remains >=5 immediately prior to randomization | 1) Age >= 18 years 2) AIS symptom onset (last known well) within 4.5 to 24 hours 3) Functionally independent (mRS 0-2) prior to stroke onset 4) Baseline NIHSS 6-25 (both included) 5) No intention to proceed to EVT |
|  | *Imaging Criteria* | | 1) ICA, MCA-M1/M2, ACA occlusion by MRA or CTA 2) Target mismatch profile on CT perfusion or MR perfusion: ischemic core volume <70 mL, mismatch ratio >1.2 and mismatch volume >10 mL | 1) ICA or MCA-M1, M2 occlusion by MRA or CTA 2) Target mismatch profile on CT perfusion or MR perfusion: ischemic core volume <70 mL, mismatch ratio >=1.8 and mismatch volume >=15 mL | 1) ICA or MCA-M1, M2 occlusion by MRA or CTA 2) Target mismatch profile on CT perfusion or MR perfusion: ischemic core volume <70 mL, mismatch ratio >=1.8 and mismatch volume >=15 mL |
|  | *Age* | *TNK* | 64.2±10.4 | 72 (62-79) | 67 (58-75) |
|  |  | *Control* | 63.6±11.0 | 73 (63-82) | 68 (59-76) |
|  | *Female sex* | *TNK* | 31 (27.9) | 122 (53.5) | 81 (30.7) |
|  |  | *Control* | 33 (29.2) | 123 (53.5) | 85 (33.7) |
|  | *Median baseline NIHSS scores* | *TNK* | 9 (5-14) | 12 (8-17) | 11 (7-15) |
|  |  | *Control* | 9 (6-16) | 12 (8-18) | 10 (7-14) |
|  | *EVT performed* | *TNK* | 58 (52.3) | 176 (77.2) | 4 (1.5) |
|  |  | *Control* | 64 (56.6) | 178 (77.4) | 5 (2.0) |
|  | *Witnessed stroke* | *TNK* | 43 (38.7) | NA | 143 (53.2) |
|  |  | *Control* | 43 (38.1) | NA | 149 (59.1) |
| **Study Procedure** | *Median time from LKW to lytic* | *TNK* | NA | 12.7 (9.2-15.8) | 12.4 (8.8-16.3) |
|  |  | *Control* | NA | 13.0 (9.0-16.9) | 12.8 (9.0-17.5) |
|  | *Study interventions* | | Tenecteplase (0.25 mg per kilogram of body weight, up to 25 mg) | Tenecteplase (0.25 mg per kilogram of body weight, up to 25 mg) | Tenecteplase (0.25 mg per kilogram of body weight, up to 25 mg) |
|  | *Study comparators* | | Best medical treatment (including Alteplase) | Placebo | Standard medical treatment |

Mean±SD, median (interquartile range), and counts (percentage) were displayed for numerical variables. TNK, tenecteplase; CHABLIS-T II, Chinese Acute Tissue-Based Imaging Selection for Lysis in Stroke Tenecteplase II; TIMELESS, Thrombolysis in Imaging-eligible, Late-window Patients to Assess the Efficacy and Safety of Tenecteplase; TRACE-III, Tenecteplase Reperfusion Therapy in Acute Ischemic Cerebrovascular Events Ⅲ; PROBE, prospective, randomized, open-label, blinded-endpoint; mRS, modified Rankin scale; NIHSS, National Institutes of Health Stroke Scale; ICA, internal carotid artery; MCA, middle cerebral artery; ACA, anterior cerebral artery; CTA, computed tomography angiography; MRA, magnetic resonance angiography; TNK, tenecteplase; EVT, endovascular thrombectomy; LKW, last known well; NA, not applicable, reflecting missing data.

**Table S2 Outcome definitions of included studies.**

| **Outcomes** | CHABLIS-T **Ⅱ** | **TIMELESS** | TRACE **Ⅲ** |
| --- | --- | --- | --- |
| **Reperfusion** | Major reperfusion without sICH occurred 24 to 48 hours: For patients without preplanned emergent EVT, major reperfusion was defined as the hypoperfusion lesion volume (DT >3 seconds) of the 4- to 6-hour repeat CTP decreased to <50% of the hypoperfusion lesion volume of the baseline CTP; For patients with preplanned emergent EVT, major reperfusion was assessed as a mTICI score 2b to 3 at the initial catheter angiography. | Reperfusion at 24 hours: more than 90% reduction in the penumbra, as estimated from the volume of tissue for which there was delayed arrival of an injected tracer agent exceeding 6 seconds, between baseline and 24-hour perfusion imaging. | Reperfusion at 24 hours: >90% reduction in the volume of the lesion in which there had been a delayed arrival of an injected tracer agent of >6 seconds, between baseline and 24-hour perfusion imaging. |
| **Recanalization** | Recanalization at 4-6 hours: defined as a score ≥2 on the Thrombolysis in Myocardial Infarction scale at the 4-hour to 6-hour CTA (reconstructed from CTP) for patients not transferred to angio-suite, or at first angiographic acquisition prior to thrombectomy for patients transferred to angio-suite. | Recanalization at 24 hours: complete recanalization (AOL score, 3; scale range, 0 [no recanalization] to 3 [complete recanalization]). | Recanalization at 24 hours: complete recanalization (AOL score, 3; scale range, 0 [no recanalization] to 3 [complete recanalization]). |
| **sICH** | sICH at 24-48 hours: any type of intracranial hemorrhage with at least four points increase in the NIHSS score from baseline. | sICH within 36 hours: an increase (indicating worsening) of at least 4 points in the NIHSS score, as compared with the most recent NIHSS score, that was attributed to bleeding on CT (preferred) or MRI performed within 36 hours after the receipt of tenecteplase or placebo. | sICH within 36 hours: the presence of any extravascular blood in the brain or within the cranium that was associated with clinical deterioration, as defined by an increase of 4 points or more in the score on the NIHSS, or that led to death and that was identified as the predominant cause of the neurologic deterioration. |
| **PH2** | Type 2 parenchymal hematoma at 24–48 hours after treatment. | Type 2 parenchymal hematoma within 72 hours. | Type 2 parenchymal hematoma within 36 hours according to ECASS criteria. PH2 represents dense blood clot(s) exceeding 30% of the infarct volume with significant space-occupying effect. |

sICH, symptomatic intracranial hemorrhage; PH2, parenchymal hematoma type 2; EVT, endovascular therapy; DT, delay time; CTP, computed tomography perfusion; mTICI, modified Thrombolysis in Cerebral Infarction; CTA, computed tomography angiography; NIHSS, National Institutes of Health Stroke Scale; AOL, Arterial Occlusive Lesion; ECASS, European Cooperative Acute Stroke Study.

**Table S3** Univariate meta-regression analyses.

| **Covariates** | **mRS 0-2 at 90 days** | **Recanalization** | **Reperfusion** |
| --- | --- | --- | --- |
| Age | 0.043 (-0.106 to 0.192) | 0.733 (-0.111 to 0.258) | -0.159 (-0.247 to -0.070) |
| Study settings | -0.066 (-1.322 to 1.189) | -0.226 (-1.193 to 1.474) | 1.014 (-0.234 to 2.264) |
| Sample size | 0.002 (0.003 to 0.005) | 0.003 (0.000 to 0.006) | -0.004 (-0.010 to 0.02) |
| Thrombectomy rates | -0.543 (-2.026 to 0.941) | -0.599 (-2.954 to 1.756) | -0.494 (-3.973 to 2.985) |
| Imaging criteria and definitions of outcomes^1^ | NA | 0.850 (0.080 to 1.620) | -1.134 (-2.385 to 0.116) |

Results were shown with coefficients and the corresponding 95% confidence intervals. mRS, modified Rankin scale score; NA, not applicable.

^1^For this variable, the CHABLIS-T II study (with its distinct imaging selection criteria and outcome definitions for recanalization and reperfusion) was assigned a value of 0, whereas the TRACE-III and TIMELESS studies were assigned a value of 1.

**Table S4** Summary of findings for efficacy outcomes.

| **Tenecteplase compared to control for acute ischemic stroke due to large vessel occlusion at 4.5 to 24 hours after last known well** | | | | | |
| --- | --- | --- | --- | --- | --- |
| **Patient or population:** acute ischemic stroke due to LVO at 4.5 to 24 hours after last known well  **Intervention:** tenecteplase  **Comparison:** placebo/standard medical treatment | | | | | |
| Outcomes | **Anticipated absolute effects^*^** (95% CI) | | Relative effect (95% CI) | № of participants (studies) | Certainty of the evidence (GRADE) |
|  | **Risk with control** | **Risk with tenecteplase** |  |  |  |
| mRS 0-1 | 274 per 1,000 | **340 per 1,000** (288 to 398) | **OR 1.36** (1.07 to 1.75) | 1195 (3 RCTs) | ⨁⨁⨁⨁ High |
| mRS 0-2 | 416 per 1,000 | **446 per 1,000** (354 to 545) | **OR 1.13** (0.77 to 1.68) | 1195 (3 RCTs) | ⨁⨁◯◯ Low^a,b^ |
| Recanalization | 284 per 1,000 | **566 per 1,000** (385 to 731) | **OR 3.30** (1.58 to 6.86) | 1092 (3 RCTs) | ⨁◯◯◯ Very low^a,b^ |
| Reperfusion | 276 per 1,000 | **416 per 1,000** (247 to 608) | **OR 1.87** (0.86 to 4.07) | 1046 (3 RCTs) | ⨁◯◯◯ Very low^a,b^ |
| ***The risk in the intervention group** (and its 95% confidence interval) is based on the assumed risk in the comparison group and the **relative effect** of the intervention (and its 95% CI).  **CI:** confidence interval; **OR:** odds ratio; LVO, large vessel occlusion; mRS, modified Rankin scale; RCT, randomized controlled trial. | | | | | |

**GRADE Working Group grades of evidence**
**High certainty:** we are very confident that the true effect lies close to that of the estimate of the effect.
**Moderate certainty:** we are moderately confident in the effect estimate: the true effect is likely to be close to the estimate of the effect, but there is a possibility that it is substantially different.
**Low certainty:** our confidence in the effect estimate is limited: the true effect may be substantially different from the estimate of the effect.
**Very low certainty:** we have very little confidence in the effect estimate: the true effect is likely to be substantially different from the estimate of effect.

Explanations

a. Substantial heterogeneity was detected.

b. A very wide range of CI was discerned.

**Table S5** Summary of findings for safety outcomes.

| **Tenecteplase compared to control for acute ischemic stroke due to large vessel occlusion at 4.5 to 24 hours after last known well** | | | | | |
| --- | --- | --- | --- | --- | --- |
| **Patient or population:** acute ischemic stroke due to LVO at 4.5 to 24 hours after last known well  **Intervention:** tenecteplase  **Comparison:** placebo/standard medical treatment | | | | | |
| Outcomes | **Anticipated absolute effects^*^** (95% CI) | | Relative effect (95% CI) | № of participants (studies) | Certainty of the evidence (GRADE) |
|  | **Risk with control** | **Risk with tenecteplase** |  |  |  |
| mRS 5-6 | 217 per 1,000 | **212 per 1,000** (168 to 262) | **OR 0.97** (0.73 to 1.28) | 1195 (3 RCTs) | ⨁⨁⨁◯ Moderate^a^ |
| Mortality | 145 per 1,000 | **151 per 1,000** (114 to 199) | **OR 1.05** (0.76 to 1.46) | 1172 (3 RCTs) | ⨁⨁⨁◯ Moderate^a^ |
| sICH | 21 per 1,000 | **36 per 1,000** (18 to 71) | **OR 1.76** (0.85 to 3.62) | 1172 (3 RCTs) | ⨁⨁⨁◯ Moderate^a^ |
| PH2 | 19 per 1,000 | **38 per 1,000** (19 to 76) | **OR 2.04** (0.98 to 4.27) | 1172 (3 RCTs) | ⨁⨁⨁◯ Moderate^a^ |
| ***The risk in the intervention group** (and its 95% confidence interval) is based on the assumed risk in the comparison group and the **relative effect** of the intervention (and its 95% CI).  **CI:** confidence interval; **OR:** odds ratio; LVO, large vessel occlusion; mRS, modified Rankin scale; sICH, symptomatic intracranial hemorrhage; PH2, parenchymal hematoma type 2; RCT, randomized controlled trial. | | | | | |

**GRADE Working Group grades of evidence**
**High certainty:** we are very confident that the true effect lies close to that of the estimate of the effect.
**Moderate certainty:** we are moderately confident in the effect estimate: the true effect is likely to be close to the estimate of the effect, but there is a possibility that it is substantially different.
**Low certainty:** our confidence in the effect estimate is limited: the true effect may be substantially different from the estimate of the effect.
**Very low certainty:** we have very little confidence in the effect estimate: the true effect is likely to be substantially different from the estimate of effect.

Explanations

1. A wide range of CI was discerned
